# Trace Amines and the Trace Amine-Associated Receptor 1: Pharmacology, Neurochemistry, and Clinical Implications

**DOI:** 10.3389/fnins.2016.00148

**Published:** 2016-04-05

**Authors:** Yue Pei, Aman Asif-Malik, Juan J. Canales

**Affiliations:** Department of Neuroscience, Psychology and Behaviour, University of LeicesterLeicester, UK

**Keywords:** trace amines, trace amine-associated receptor 1, Parkinson's disease, schizophrenia, mood disorders, addiction

## Abstract

Biogenic amines are a collection of endogenous molecules that play pivotal roles as neurotransmitters and hormones. In addition to the “classical” biogenic amines resulting from decarboxylation of aromatic acids, including dopamine (DA), norepinephrine, epinephrine, serotonin (5-HT), and histamine, other biogenic amines, present at much lower concentrations in the central nervous system (CNS), and hence referred to as “trace” amines (TAs), are now recognized to play significant neurophysiological and behavioral functions. At the turn of the century, the discovery of the trace amine-associated receptor 1 (TAAR1), a phylogenetically conserved G protein-coupled receptor that is responsive to both TAs, such as β-phenylethylamine, octopamine, and tyramine, and structurally-related amphetamines, unveiled mechanisms of action for TAs other than interference with aminergic pathways, laying the foundations for deciphering the functional significance of TAs and its mammalian CNS receptor, TAAR1. Although, its molecular interactions and downstream targets have not been fully elucidated, TAAR1 activation triggers accumulation of intracellular cAMP, modulates PKA and PKC signaling and interferes with the β-arrestin2-dependent pathway via G protein-independent mechanisms. TAAR1 is uniquely positioned to exert direct control over DA and 5-HT neuronal firing and release, which has profound implications for understanding the pathophysiology of, and therefore designing more efficacious therapeutic interventions for, a range of neuropsychiatric disorders that involve aminergic dysregulation, including Parkinson's disease, schizophrenia, mood disorders, and addiction. Indeed, the recent development of novel pharmacological tools targeting TAAR1 has uncovered the remarkable potential of TAAR1-based medications as new generation pharmacotherapies in neuropsychiatry. This review summarizes recent developments in the study of TAs and TAAR1, their intricate neurochemistry and pharmacology, and their relevance for neurodegenerative and neuropsychiatric disease.

## Introduction

The classic biogenic amines, including dopamine (DA), norepinephrine, epinephrine, serotonin (5-HT), and histamine, are neurotransmitters that play vital roles in the regulation of a wide variety of neurophysiological, behavioral, and cognitive processes such as motor control, affect, motivation, learning, and memory. Alterations in aminergic transmission have been extensively documented in a broad range of neurological disorders including Parkinson's disease (PD), schizophrenia, attention deficient hyperactivity disorder (ADHD), depression and drug addiction, amongst others. Accordingly, the mainstay pharmacological approach to the treatment for such conditions has by and large relied on directly altering neurotransmission of these classic monoamines. However, limited success has been achieved thus far in the symptomatic relief in these disorders, with direct manipulation of such neurotransmitter systems being in some instances associated with untoward side effects. In this regard, the DA system is a prototypical example. Chronic L-DOPA treatment remains superior to other DA receptor agonist-based interventions as symptomatic treatment for PD but leads to long-term, debilitating motor complications such as on-off fluctuations and dyskinesias (Kostic et al., 1991; Fahn, 2000; Fabbrini et al., 2007; Kalia et al., 2013). Similarly, neuroleptics, which are used for the treatment of psychotic and bipolar disorder symptomatology, can cause extrapyramidal side-effects characterized by acute dystonia, akathisia, parkinsonism, and tardive dyskinesia (Ghaemi et al., 2006; Gao et al., 2008). Moreover, the DA transporter (DAT) has long been targeted to develop substitution treatments for drug addiction. However, progress has been hindered by the potential abuse liability and non-specific effects associated with replacement medications based on DAT pharmacology, although promising results with long-acting compounds have been reported (Desai et al., 2005; Rothman et al., 2008; Ferragud et al., 2009; Tanda et al., 2009a,b; Velazquez-Sanchez et al., 2009, 2010; Velázquez-Sánchez et al., 2013; Reith et al., 2015). During the on-going search for more efficacious medications in neuropsychiatry, the trace amine (TA) transmitter system, a secondary amine system that is intimately related to the classic biogenic amines, has attracted increasing attention in recent years. TAs belong to a group of endogenous amines whose neurobiological functions were largely neglected until the breakthrough discovery of a group of G protein-coupled receptors (GPCRs), the so-called trace amine-associated receptors (TAARs), by two independent research groups in 2001 (Borowsky et al., 2001; Bunzow et al., 2001). Research conducted in the past 15 years has concentrated primarily on TAAR1, as the only member of this family of receptors that is both responsive to TAs and phylogenetically conserved in the mammalian brain (Borowsky et al., 2001; Lindemann et al., 2005). TAAR1 has proved to be an important modulator of the major biogenic amines and has begun to gain accumulated support as a promising target for medicinal development in a variety of neuropsychiatric disorders. In the current review we summarize the latest advancements in understanding the role of the TA system in the mammalian central nervous system (CNS), focusing on its pharmacology and neurochemistry and on the important implications of TAAR1 in the pathophysiology and treatment of neuropsychiatric disorders that involve aminergic dysregulation.

## Trace amines

TAs, including ρ-tyramine, *m*-tyramine, β-phenylethylamine (β-PEA), *m*-octopamine, ρ-octopamine, tryptamine, and synephrine are a group of endogenous amines found in both invertebrate and vertebrate species (Berry, 2004). TAs have well-documented roles in invertebrates as major neurotransmitters, with octopamine believed to be the sympathetic nervous system counterpart of norepinephrine in vertebrates (Robertson and Juorio, 1976; Evans and O'Shea, 1977; Roeder, 1999). By contrast, although the existence of TAs in the vertebrate brain and peripheral nervous system has long been recognized, their functions were largely unknown due to the apparent lack of identified receptors specific for TAs, which prompted their description as “false neurotransmitters” (Berry, 2004). TAs are structurally similar to, and share same biosynthetic and metabolic pathways with, the classic monoamines (Berry, 2004; Ledonne et al., 2011). Initial studies postulated that TAs exerted sympathomimetic actions in the vertebrate peripheral nervous system, linking them to blood pressure regulation and electrolyte homeostasis (Barger and Dale, 1910; Podder et al., 1979). This notion can be traced back to the clinical observation of the so-called “cheese reaction,” a hypertensive crisis experienced by sensitive patients treated with monoamine oxidase (MAO) inhibitor class of antidepressant drugs and exposed to aged cheese and other types of processed food enriched in ρ-tyramine produced through bacteria decarboxylation during fermentation (Blackwell and Mabbitt, 1965; Boulton et al., 1970; Rice et al., 1976; Stratton et al., 1991; Anderson et al., 1993). It has been shown that peripheral tyramine releases endogenous norepinephrine from peripheral stores, which in turn stimulates adrenergic nerves, a process responsible for its indirect sympathetic action (Crout et al., 1962; Tapper et al., 1981).

In the CNS, TAs are present at low nanomolar concentrations at a range that is several hundred-fold below that of the classical neurotransmitters, which can be linked to their extremely rapid turnover rate and a very short half-life of around 30 s (Burden and Philips, 1980; Berry, 2004). TAs exhibit a heterogeneous distribution that closely parallels the classic monoaminergic projection pathways, with enhanced expression in the nigrostriatal and mesolimbic dopaminergic pathways (Philips, 1984). Evidence suggests that some TAs, including ρ-tyramine, β-PEA, and tryptamine, are synthesized within nigrostriatal DA neurons while ρ-octopamine is synthesized within adrenergic neurons (Berry, 2004). However, as indicated previously, TAs were regarded for a considerable time as mere metabolic by-products of other neurotransmitters and having little neurophysiological significance in their own right. In subsequent studies, TAs were classified as endogenous neuromodulators that regulated, and were themselves susceptible to regulation by, co-existing neurotransmitters (Berry, 2004). Indeed, ample evidence has demonstrated an intimate functional inter-regulation between TAs and the classic monoamines, especially DA. First, changes in monoamine activity are able to alter TA levels. For example, while reductions in striatal levels of β-PEA (Juorio et al., 1991) and ρ-tyramine (Jones et al., 1983) were found to increase DA release triggered by electrical stimulation of the substantia nigra, inhibition of DA neurotransmission led to an elevated accumulation rate of β-PEA in the striatum (Juorio et al., 1991). Reciprocally, TAs appeared to potentiate the efficacy of synaptic transmission mediated by these monoamines (Philips, 1984; Burchett and Hicks, 2006). For instance, administration of MAO-B inhibitors, which increased β-PEA levels above their physiological range, enhanced the striatal neuronal response to DA (Berry et al., 1994) and DA agonists (Paterson et al., 1991). Moreover, iontophoretic application of β-PEA elicited a potentiated cortical neuronal response to norepinephrine (Paterson and Boulton, 1988; Paterson, 1993). Similarly, iontophoretic ejection of ρ-tyramine, *m*-tyramine, and β-PEA applied at weak currents increased the cortical neuronal response to DA (Jones and Boulton, 1980). Likewise, application of octopamine through weak iontophoretic currents enhanced both inhibitory and excitatory neuronal response mediated by norepinephrine (Jones, 1982a). The effect of tryptamine on 5-HT neurotransmission appeared to be more complex as both a depression and potentiation of 5-HT-mediated neuronal effects were observed, which might be accounted for by the biphasic effects of 5-HT itself on cortical neuron activity (Jones, 1982b). Taken together, these findings suggest that TAs are likely to serve as a fine-tuning mechanism that keeps a balanced monoaminergic tone by responding to endogenous- or exogenous-induced monoamine fluctuations, a process that may be partly mediated through interaction with specific receptors for TAs (Berry, 2004).

Given the abovementioned reciprocal relationship between TAs and monoamines, it is not surprising that dysfunction of TA signaling has been historically associated with an ample spectrum of neurological pathologies that involve changes in monoamine function. For example, a reduced level of urinary excretion of β-PEA was found in children with ADHD (Baker et al., 1991). Moreover, while deficient synthesis of two TAs, tyramine, and octopamine (Sandler et al., 1979), and decreased urinary excretion of β-PEA (Wolf and Mosnaim, 1983), have been found in patients with depressive disorders, PEA replacement treatment produced long-lasting relief of depression in a patient population (Sabelli et al., 1995). Furthermore, women with bipolar affective disorder show very high rate of urinary β-PEA excretion whilst MAO inhibitors, which further increased β-PEA excretion, exacerbated their symptoms (Karoum et al., 1982). In addition, chronic treatment with the psychotogenic drugs, lysergic acid diethylamide (LSD) and phencyclidine, produced upregulation of aromatic L-amino acid decarboxylase (AADC, an enzyme that catalyses the synthesis of β-PEA) mRNA levels, indicating that over-production of β-PEA may be involved in the pathogenesis of psychotic syndromes (Buckland et al., 1997). Indeed, urinary β-PEA level was found to be significantly elevated in paranoid chronic schizophrenics (Potkin et al., 1979).

TAs have also been implicated in the action of psychostimulants and, more widely, in drug addiction. For example, β-PEA bears close structural and pharmacological similarity with the central stimulant, amphetamine (Tinklenberg et al., 1978; Janssen et al., 1999). At concentrations that were several orders of magnitude above its normal physiological range, β-PEA induced amphetamine-like effects in rodents and monkeys, including hyperactivity (Dourish, 1985) and stereotypic behavior (Borison et al., 1977; Tinklenberg et al., 1978). This evidence led to the characterization of β-PEA as brain “endogenous amphetamine” (Janssen et al., 1999). Moreover, β-PEA activity is altered by exogenous application of *d*-amphetamine. Acute application of *d*-amphetamine resulted in an initial decrease and a subsequent increase in brain level of β-PEA in rabbits (Borison et al., 1975). Also, chronic administration of amphetamine in rats downregulated AADC mRNA levels, which could in turn lead to decreased β-PEA activity (Buckland et al., 1996). On the other hand, certain behavioral effects of amphetamine appeared to be dependent on β-PEA levels as depletion of brain β-PEA blocked the motor-stimulating effect of *d*-amphetamine in mice and rabbits (Borison et al., 1975).

Furthermore, evidence has accrued on the ability of TAs to modulate brain reward (i.e., the subjective experience of pleasure) and reinforcement (i.e., the strengthening of a conditioned response by a given stimulus; Greenshaw, 1984), suggesting the involvement of the TAs in the neurological adaptations underlying drug addiction, a chronic relapsing syndrome characterized by compulsive drug taking, inability to control drug intake and dysphoria when access to the drug is prevented (Koob, 2009). Consistent with its hypothesized role as “endogenous amphetamine,” β-PEA was shown to possess reinforcing properties, a defining feature that underlies the abuse liability of amphetamine and other psychomotor stimulants. β-PEA was also as effective as amphetamine in its ability to produce conditioned place preference (i.e., the process by which an organism learns an association between drug effects and a particular place or context) in rats (Gilbert and Cooper, 1983) and was readily self-administered by dogs that had a stable history (i.e., consisting of early acquisition and later maintenance) of amphetamine or cocaine self-administration (Risner and Jones, 1977; Shannon and Thompson, 1984). In another study, high concentrations of β-PEA dose-dependently maintained responding in monkeys that were previously trained to self-administer cocaine, and pretreatment with a MAO-B inhibitor, which delayed β-PEA deactivation, further increased response rates (Bergman et al., 2001). However, other studies have revealed an opposing action of TAs on brain reward. In rats that responded at threshold levels for intracranial self-stimulation (ICSS) of the lateral hypothalamus, tryptamine antagonists not only caused dose-related increases when given alone but also potentiated the facilitating effects of amphetamine (Silveira Filho and Graeff, 1977). Conversely, systematic application of tryptamine decreased intracranial self-stimulation (ICSS) in both the medial raphe nucleus and the lateral hypothalamus (Broadbent and Greenshaw, 1985). These findings suggest an inhibitory regulation of brain reward processing via the tryptamine-mediated pathway. Also, in sharp contrast to the conditioned place preference produced by β-PEA, as noted above, tryptamine induced conditioned taste aversion to a novel saccharin solution in rats (Fletcher, 1986), suggesting a negative influence on reward-related behavior. Moreover, while little is known about the role of octopamine in brain reward in vertebrates, the functional contribution of octopamine to reward-associated learning has been well-documented in insects (Hammer, 1997; Unoki et al., 2006; Perry and Barron, 2013). Studies conducted in *Drosophila* suggest that tyramine is essential for the development of cocaine sensitization (McClung and Hirsh, 1999), a phenomenon thought to share similar underlying neuroadaptive mechanisms to those mediating craving and relapse (Kalivas et al., 1998).

Although these findings have underscored the implication of TAs in key behavioral and neurological functions, the signaling mechanisms and downstream molecular targets to which they are coupled have remained unclear until recently. For a considerable time, the prevailing view regarding the mode of action of TAs held two possible routes of action (Sotnikova et al., 2004; Burchett and Hicks, 2006). First, it was suggested that TAs interact with plasma membrane transporters to inhibit monoamine uptake and induce efflux through reverse transport or interfere with monoamine vesicular storage to displace the classic monoamines from their storage pool (Raiteri et al., 1977; Parker and Cubeddu, 1988). Second, TAs could bind to yet unidentified TA-sensitive signaling proteins located on pre- or post-synaptic neurons containing GPCRs for the classic monoamines, thereby modulating their corresponding intracellular second messenger pathways (Premont et al., 2001; Sotnikova et al., 2004; Burchett and Hicks, 2006). Moreover, in addition to altering the major aminergic pathways, TAs have been shown to modulate neuronal signaling mediated by other important neurotransmitters, such as gamma-aminobutyric acid (GABA; Berretta et al., 2005; Federici et al., 2005) and acetylcholine (Kato et al., 2001; Ishida et al., 2005), but the functional relevance of such interactions is not well-understood at present.

## Identification of TAAR family

Progress in the characterization of the neurobiological functions of TAs has been hampered by the difficulty in identifying their specific receptor targets and the lack of selective agonists and antagonists for such receptors. Although, saturable high-affinity binding sites distinct from the amine transporters and receptors had been identified in the mammalian brain (Kellar and Cascio, 1982; Brüning and Rommelspacher, 1984; McCormack et al., 1986; Nguyen and Juorio, 1989), it was at the beginning of the twenty-first century that two research groups independently reported the cloning and identification of a novel family of mammalian GPCRs (Borowsky et al., 2001; Bunzow et al., 2001). Such receptors, including several orphan receptors, shared an unusually high degree of sequence homology and some were directly activated by TAs. The discovery of receptors for TAs supported their role as *bona fide* neurotransmitters, this is, as molecules able to trigger cellular events directly, and led to a renewed interest in the TAs and their biological functions. In subsequent studies, Lindemann and collaborators proposed a uniform nomenclature for this newly discovered GPCR family, together with closely related receptors, as trace-amine-associated receptors (TAARs), acknowledging the fact that some members are unresponsive to TAs (Lindemann et al., 2005). Further, work by the same group completed the identification of all members of this GPCR family in rats, mice, chimpanzees, and humans, demonstrating remarkable differences in the number of receptor genes and the proportion of pseudogenes amongst the four species (Lindemann et al., 2005). There are nine *TAAR* genes in human including three pseudogenes; nine genes in chimpanzee including six pseudogenes; 19 and 16 in rat and mouse with two and one being pseudogenes, respectively. In spite of these significant inter-species differences, three TAAR subfamilies were identified based on phylogenetic relationships and pharmacophore similarities, which remained consistent across all of the four species (Lindemann et al., 2005). The three subfamilies consist of TAARs 1–4, TAAR5, and TAARs 6–9, with each subgroup represented by at least one functional *TAAR* gene. Not surprisingly, the only two receptors that are activated by TAs, namely TAAR1 and TAAR4, both belong to the first subfamily, supporting a functional basis for the classification (Lindemann et al., 2005). While TAAR1 is sensitive to all TAs and the *TAAR1* gene is phylogenetically conserved in all the studied species including human, *TAAR4* is a pseudogene in the human genome and rat TAAR4 responds only to β-PEA and tyramine, although to a much lesser degree than TAAR1 (Borowsky et al., 2001; Bunzow et al., 2001; Lindemann et al., 2005). As a result, TAAR1 has received by far the most attention over the past decade and it is the best characterized receptor of the class (Lindemann and Hoener, 2005).

## Expression, signaling, and pharmacology of TAAR1

**Figure 1:**
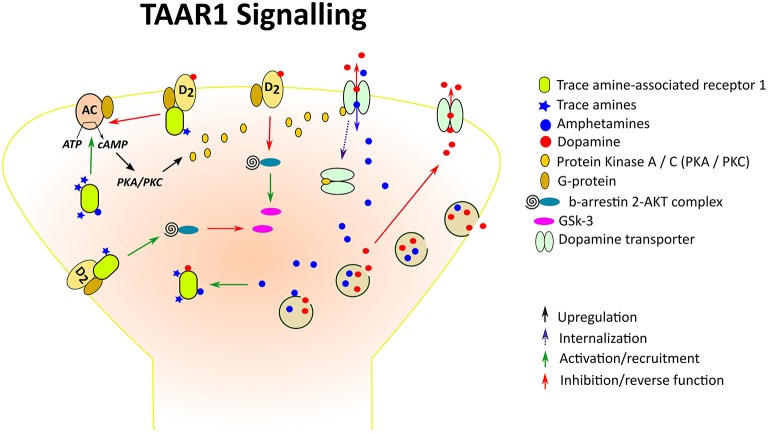
**Trace amine-associated receptor 1 (TAAR1), a TA- and amphetamine-activated GPCR, at a DA synapse**. Amphetamines enter the presynaptic neurons through competitive reuptake inhibition of the DA transporter (DAT) and by diffusion through presynaptic membranes, causing release of DA by way of vesicular monoamine transporter-mediated exocytosis and reverse transport through the DAT. Both amphetamines and endogenous TAs activate TAAR1, leading to adenylyl cyclase (AC) activation and downstream stimulation of PKA/PKC. Like D2R, TAAR1 also signals via a G-protein independent, β-arrestin2-dependent pathway involving the protein kinase B (AKT)/glycogen synthase kinase 3 (GSK-3) β signaling cascade. In the case of striatal D2R, β-arrestin 2 has been shown to enhance the interaction between AKT and protein phosphatase 2A thereby resulting in AKT inactivation and increased activation of GSK-3. D2s are also GPCRs which when attached to TAAR1 form heterodimeric complexes. Interaction of TAAR1 with D2R reduces β-arrestin 2 recruitment to D2R and the ability of D2R to decrease cAMP production, whereas β-arrestin 2 signaling is enhanced, resulting in reduced GSK-3 activation. In addition, phosphorylation of the DAT through TAAR1-stimulated activation has been shown to lead to DAT internalization, which may result in reduced DA uptake under certain conditions.

TAAR1 couples to a Gα_*s*_ G protein and, upon stimulation, triggers accumulation of intracellular cAMP via adenylyl cyclase activation and stimulates inwardly rectifying K^+^ channels (Borowsky et al., 2001; Bunzow et al., 2001; Miller et al., 2005; Xie et al., 2007; Bradaia et al., 2009). TAAR1 activation can also lead to PKA and PKC phosphorylation and upregulation of the transcription factors, CREB and NFAT (Panas et al., 2012). TAAR1 also signals via a G-protein independent, β-arrestin2-dependent pathway involving the protein kinase B (AKT)/glycogen synthase kinase 3 (GSK-3) β signaling cascade, an important player in many DA-mediated actions (Harmeier et al., 2015; Figure 1). Among the TAs, β-PEA and tyramine are the most potent activators at TAAR1, with β-PEA being more potent than tyramine at human and mouse TAAR1, with the opposite being true at rat TAAR1 (Bunzow et al., 2001; Grandy, 2007; Wainscott et al., 2007). Strikingly, in addition to TAs as principal binding ligands, TAAR1 is also activated by a vast variety of endogenous and exogenous molecules, including the major catecholamines, DA, norepinephrine, and 5-HT and some of their metabolites, amphetamine-like compounds including amphetamine itself, methamphetamine and 3,4-methylenedioxymethamphetamine (MDMA), ergot derivatives including LSD, and several adrenergic ligands (Bunzow et al., 2001), as well as certain thyroid hormones derivatives (Hart et al., 2006). Studies aiming at determining TAAR1 distribution in the mammalian system have consistently reported a widespread and unique pattern of TAAR1 mRNA or protein expression in the central and peripheral nervous system in human, mouse, rat, and rhesus monkey. In the brain, TAAR1 mRNA has been detected throughout the limbic system and in regions associated with the major monoaminergic pathways, including the ventral tegmental area (VTA), substantia nigra (SNr), locus coeruleus, raphe nucleus, caudate nucleus, putamen, nucleus accumbens (NAc), hippocampus, hypothalamus, and amygdala (Borowsky et al., 2001; Bunzow et al., 2001; Miller et al., 2005; Xie et al., 2007; Lindemann et al., 2008; Espinoza et al., 2015). In addition to subcortical areas, TAAR1 is also expressed in cortical regions, especially in layer V pyramidal neurons of the prefrontal cortex (PFC; Espinoza et al., 2015). The cellular distribution of TAAR1 is predominantly intracellular, with diffuse expression within the perikaryon and along axonal processes, and sparse membrane-associated neuronal expression (Bunzow et al., 2001; Xie et al., 2007). Therefore, it has been postulated that the intracellular TAAR1 might recruit an accessory protein for translocation to the plasma membrane (Bunzow et al., 2001) or indeed might signal intracellularly, given the accessibility of several TAAR1 endogenous ligands within the cytoplasm and the ability of intracellular GPCRs to exert downstream effects (Xie et al., 2007; Lam et al., 2015). Remarkably, TAAR1 is colocalized with DAT in a subset of DA neurons, and/or expressed in neurons that are in close apposition to DAT-expressing neurons in mouse and rhesus monkey substantia nigra (Xie et al., 2007). There is also evidence suggesting co-expression of TAAR1 with the norepinephrine transporter in adrenergic neurons in the rhesus monkey locus coeruleus, as well as co-localization with 5-HT transporter in serotonergic neurons in the mouse dorsal raphe nucleus (Lindemann et al., 2008; Xie et al., 2008). The neuroanatomical distribution of TAAR1 in relation to the major monoamine systems suggests that TAAR1 might be in a position to regulate monoaminergic transmission through direct interactions with monoamine transporters and presynaptic autoreceptors co-expressed with TAAR1 within single neurons, or by way of intercellular communication with nearby monoaminergic neurons. In the next section we will review evidence from different research lines in support of such fundamental neurochemical interactions.

## Functional interaction of TAAR1 with brain monoamine systems

Studies with heterologous expression systems and brain synaptosomes have revealed a complex tripartite relationship between TAAR1, monoamine transporters, and monoamine autoreceptors *in vitro*. First, in such *in vitro* preparations, TAAR1 activation by agonist ligands, including TAs, the classic biogenic amines, and drugs of the amphetamine-class, was shown to be markedly enhanced by co-transfecting TAAR1 with monoamine transporters (Miller et al., 2005; Xie et al., 2007). Since, these TAAR1 agonists are also substrates at monoamine transporters, it has been hypothesized that the monoamine transporters might serve as conduits for the entry of TAAR1 agonists into the synaptic terminal such that activation of intracellular TAAR1 can occur (Miller et al., 2005; Xie et al., 2007; Miller, 2011). Alternatively, the transporter-mediated agonist uptake might trigger trafficking of TAAR1 into the plasma membrane (Xie et al., 2007). Additionally, such *in vitro* assays have revealed that TAAR1 activation functionally downregulates the activity of monoamine transporters. In cells co-transfected with both TAAR1 and one of the main aminergic transporters, TAAR1 activation by DA, norepinephrine or 5-HT led to functional inhibition of the co-expressed transporter, reducing uptake, and increasing efflux of the associated neurotransmitter (Xie et al., 2008).

Moreover, further evidence suggests that both TAAR1 and monoamine autoreceptor activation modulate monoamine transporter function reciprocally by way of opposing interactions on aminergic transmission (Xie et al., 2008). While TAAR1 activation promotes efflux of monoamines through their transporter proteins, autoreceptor activation leads to increases in the uptake of classical biogenic amines in monkey and wild-type mouse striatal and thalamic synaptosomes, with this effect being absent in synaptosomes from TAAR1 knockout (KO) mouse (Xie et al., 2008). The same authors showed that when the DA, norepinephrine and 5-HT transporters were co-transfected with D2s, α_2A_, or 5-HT_1B_ autoreceptors, uptake of the respective amine transmitter was significantly enhanced. Similarly, norepinephrine and 5-HT also reduced retention of the preloaded neurotransmitter in the presence of the specific autoreceptor antagonist in both monkey and wild-type mouse, but not in TAAR1 KO, synaptosomes. TAAR1-dependent monoamine efflux has been attributed to reversed transport of monoamines through their corresponding transporters resulting from TAAR1-mediated intracellular cAMP accumulation and substrate phosphorylation (Xie et al., 2008). Together, these findings indicate that the classical biogenic amines interact with both TAAR1 and monoamine autoreceptors to regulate transporter function. Thus, a concept of presynaptic receptor balancing has been proposed whereby TAAR1 and monoamine autoreceptors equilibrate monoamine activity, with the former inhibiting uptake and the latter facilitating it (Xie et al., 2008; Xie and Miller, 2009b).

More recent evidence from both *in vitro* and *in vivo* studies suggests a direct interaction of TAAR1 with monoamine autoreceptors that may underlie the presynaptic receptor balancing previously proposed. First, TAAR1 and D2s receptors, when co-expressed in cells, were able to form constitutive heterodimers in plasma membrane, thus allowing functional regulation of these two GPCR and/or other cellular substrates (Espinoza et al., 2011). Indeed, both total and membrane expression level of TAAR1 was decreased by co-expressing it with D2s in the same cells (Espinoza et al., 2011). Such interactions between TAAR1 and D2s receptors are in part mediated by receptor heterodimerization. Previously, it was shown that co-transfecting D2s, α_2*A*_, or α_2*B*_, or 5-HT_1A_ or 5-HT_1B_ along with TAAR1 attenuated TAAR1 activation-induced intracellular cAMP in response to DA, norepinephrine or 5-HT, respectively (Xie et al., 2008). Thus, the facilitating effects of autoreceptor activation on monoamine transporter function could result from either direct enhancement or suppression of TAAR1-mediated inhibition of transporter function. It is important to note that more recent studies indicate that TAAR1 activation may also potentiate D2s-mediated inhibition of monoamine transmission, an effect that is lacking in TAAR1 KO mice (Leo et al., 2014). Similarly, previous findings indicated that activating TAAR1 with specific agonists increased agonist potency at 5-HT_1A_ receptors whereas selective blockade of TAAR1 produced the opposite effects (Revel et al., 2011). Collectively, these findings suggest that TAAR1 stimulation may have dual effects on monoaminergic activity. While TAAR1's direct inhibition of the monoamine transporter may result in extracellular monoamine accumulation, TAAR1-mediated upregulation of monoamine autoreceptors may lead to enhanced transporter function and depressed monoamine transmission. However, it is important to bear in mind that controversy exists as to the existence of functional *in vivo* receptor couplings and caution should be taken when assessing the findings on *in vitro* heterodimerization and its functional consequences. For example, whilst hetero-oligomers of G-protein-coupled receptors have become the subject of much research due to their potential ability to signal differently from their component receptors, which could have implications for the development of novel pharmacotherapies, the identification of such complexes *in vivo* has been challenging (Frederick et al., 2015). Thus the validity of this approach needs to be carefully considered.

Therefore, it would appear that the relative activation of TAAR1 and D2s receptors by endogenous or exogenous ligands critically determines the net output of monoaminergic systems through key effects on transporter regulation (Xie and Miller, 2009b). Unlike the common biogenic amines, which activate both types of receptors, the endogenous TAs are agonists at TAAR1 only. Consequently, selective TAAR1 stimulation by TAs or specific agonists is distinct from TAAR1 activation by classical monoamines in that these trigger inhibitory modulation of TAAR1 through autoreceptor co-stimulation. Not surprisingly, *in vitro*, the co-expression of monoamine autoreceptors with TAAR1 attenuated TAAR1 signaling in response to common monoamines, but not to β-PEA (Xie and Miller, 2008). In agreement with these findings, while the common biogenic amines significantly enhanced uptake in cells co-transfected with the respective monoamine autoreceptors and transporters, β-PEA did not (Xie and Miller, 2008).

Further, accentuating the complexity of TAAR1 molecular interactions at monoamine synapses, recent studies have unveiled regulatory effects of TAAR1 signaling on post-synaptic D2 receptors. While TAAR1 KO mice had impaired striatal presynaptic D2s-mediated autoinhibition (Leo et al., 2014), they also exhibited upregulation of striatal post-synaptic D2 receptors mRNA and overactivity of D2 receptor-mediated G protein/cAMP-independent, β-arrestin2-dependent signaling pathway (Espinoza et al., 2015). D2 receptor activation through β-arrestin2 has been shown to dephosphorylate AKT and its downstream target GSK3β, leading to inhibited AKT activity and subsequent increase in GSK3β signaling (Beaulieu et al., 2011). Decreased phosphorylation of AKT with concomitant elevation of GSK3β is associated with excessive dopaminergic stimulation, as produced either by indirect DA agonists including psychostimulants, such as amphetamine and cocaine, or by DAT deletion (Beaulieu et al., 2004; Li and Gao, 2011). Conversely, pharmacological or genetic inhibition of GSK3β reversed DA-stimulated behaviors and reduced the effects of psychostimulants (Beaulieu et al., 2004, 2005; Li and Gao, 2011). Therefore, the finding that striatal D2 receptors and associated AKT/GSK3β signaling pathway are upregulated in TAAR1 KO mice suggests that TAAR1 activation may counteract DA signaling at post-synaptic sites, although this effect is likely to be indirect. A recent study reported that the formation of heteromeric complexes between TAAR1 and D2 receptors not only reduces TAAR1-stimulated cAMP accumulation, but also shifts β-arrestin2 recruitment from activated D2 receptors to activated TAAR1 (Harmeier et al., 2015). Thus this complementary evidence suggests that TAAR1 may be able to downregulate DA transmission not only by potentiating D2s-mediated presynaptic autoinhibition but also through inhibiting D2 receptor-mediated postsynaptic signaling. In support of this notion, mice with TAAR1 depletion exhibited greater locomotor activity compared to wild-type counterparts when challenged with quinpirole, a D2-like receptor agonist that is known to inhibit locomotion at low doses via stimulating presynaptic D2s and enhance locomotion at high doses by activating post-synaptic D2-like receptors, suggesting that TAAR1 deletion may induce super-sensitivity of post-synaptic D2-like receptors (Espinoza et al., 2015).

Taken together, these findings suggest that multidirectional interactions occur between TAAR1 and monoamine molecular targets at both pre- and post-synaptic sites. However, knowledge derived from evidence at cellular and molecular levels raises the question as to the ultimate functional outcome of these complex interactions and the exact neurophysiological role of TAAR1. Inspection of the neurological and behavioral adaptations exhibited by transgenic mice with TAAR1 modifications provides additional insight into this question. Compared to wild-type littermates, TAAR1 KO mice exhibited no differences in general health indicators as well as general motor function (Wolinsky et al., 2007; Lindemann et al., 2008) but displayed deficits in pre-pulse inhibition of acoustic startle, a DA-dependent response, indicating impaired sensorimotor gating (Wolinsky et al., 2007). Moreover, these KO mice had elevated spontaneous firing frequency and depolarized resting membrane potential of DA neurons in the VTA (Lindemann et al., 2008), amplified spontaneous spike rate of 5-HT neurons in the dorsal raphe nucleus (Revel et al., 2011), and increased extracellular DA in the NAc (Leo et al., 2014). Likewise, they showed enhanced sensitivity to amphetamine-induced locomotor activity and increased extracellular DA, norepinephrine, and 5-HT levels in the striatum (Wolinsky et al., 2007; Lindemann et al., 2008), and methamphetamine-induced conditioned place preference (Achat-Mendes et al., 2012). When challenged with MDMA (“ecstasy”), the wild-type mice displayed dose-dependent, biphasic thermoregulatory responses with early hypothermia followed by hyperthermia, but TAAR1 KO mice only showed long-lasting hyperthermia accompanied by supersensitivity to MDMA-stimulated locomotor activity and release of DA and 5-HT in the NAc and dorsal striatum, and of DA in the frontal cortex (Di Cara et al., 2011).

By contrast, mice engineered to overexpress TAAR1 showed unaltered spontaneous locomotor activity but hyposensitivity to amphetamine-induced psychomotor activity and catecholamine release in the NAc (Revel et al., 2012a). Taken together, these findings suggest that TAAR1 may be constitutively active or tonically activated by ambient levels of endogenous amines to exert an inhibitory influence on monoaminergic neurotransmission. Furthermore, these data suggest that TAAR1 activation may reduce the potentiation of monoaminergic transmission elicited by motor stimulant drugs. TAAR1 activation by amphetamine, methamphetamine and MDMA, which are themselves agonists at TAAR1, may reduce the neurochemical and behavioral actions of these drugs (Lindemann et al., 2008; Di Cara et al., 2011; Miller, 2011).

Studies with the most recently engineered highly selective TAAR1 agonists and antagonists have provided more direct evidence for TAAR1's inhibitory influence on monoamine transmission. The selective TAAR1 antagonist, EPPTB, increased the firing rate of DA neurons in mouse VTA (Bradaia et al., 2009), which is in agreement with the notion that TAAR1 is constitutively active or tonically activated by ambient levels of amines to downregulate DA activity. Moreover, while the selective TAAR1 full agonists, RO5166017 and RO5256390, decreased the firing frequency of DA neurons in the VTA and of 5-HT neurons in the dorsal raphe nucleus, the partial agonists, RO5203648 and RO5263397, enhanced the firing rate of these same neurons, further suggesting high constitutive activity or tonic activation of TAAR1, that serves to keep monoamine transmission in balance. These findings have led to the suggestion that pharmacological TAAR1 activation may provide a means to modulate altered DA transmission in pathophysiological states (Revel et al., 2011, 2012a, 2013). Subsequent studies have reported findings in agreement with this possibility. Whereas the full agonist, RO5166017, reduced electrically evoked DA release in both the dorsal striatum and NAc in slices of mouse brain, the antagonist, EPPTB, increased NAc DA release (Leo et al., 2014). Further, the partial agonist, RO5203648, prevented cocaine-induced DA-overflow, *in vitro*, (Pei et al., 2014) and transiently attenuated methamphetamine-induced DA accumulation in the NAc, *in vivo* (Cotter et al., 2015), although it increased the firing rate of DA neurons in the VTA under basal conditions. In behavioral experiments, both full and partial agonists blocked the hyperlocomotion induced by cocaine or amphetamine in wild-type mice and mutant mice with genetic DAT deletion (Revel et al., 2011, 2012a, 2013). Collectively, these findings strongly support a role for TAAR1 as a regulator or stabilizer of the DA system.

## TAAR1 ligands and implications

The discovery of TAAR1 as a distinguishable binding site for TAs not only supported the categorisation of TAs as *bona fide* neurotransmitters but also unveiled a potential underlying mechanism linking various neuropsychiatric disorders with dysfunction of the TA system. As discussed above, TAAR1 activation by TAs can regulate monoamine transporter and monoamine transmission, supporting TAAR1 as the action site through which TAs exert control on neurophysiological processes (Xie and Miller, 2008). However, caution should be exerted when attributing TAs' effects to TAAR1 because routes of action other than TAAR1 may also exist for TAs (Van Nguyen et al., 1989; Vaccari, 1993; Borowsky et al., 2001). For example, evidence has revealed that β-PEA and tyramine reversibly reduced D2s autoreceptor-mediated G-protein-gated inward rectifier K^+^ channel currents in mice midbrain DA neurons by acting at sites other than TAAR1 (Ledonne et al., 2010). Additionally, several by-products of catecholamine metabolism exert actions at TAAR1. 3-methoxytyramine, normetanephrine, and metanephrine, the meta-O-methyl metabolites of DA, norepinephrine, and adrenaline, respectively, cause TAAR1-stimulated cAMP elevations with significantly higher potencies than those achieved by their parent catecholamine (e.g., 3-MT vs, DA—2-fold; (±)NMN vs norepinephrine (0.1)—4-fold; (±)MN vs. epinephrine—2.5-fold; Bunzow et al., 2001). Given that these metabolites are generally considered as biologically inactive degradation products, the discovery of their high binding affinity at TAAR1 suggests that their importance may be greater than previously recognized. Furthermore, the direct DA agonists apomorphine and bromocriptine were found to activate TAAR1 *in vitro* (Bunzow et al., 2001). Although, the degree to which their possible interaction with TAAR1 contributes to their physiological and behavioral effects *in vivo* remains speculative, recent evidence suggests that certain characteristic stereotypic behaviors, such as climbing and licking, induced by high doses of apomorphine, are at least partially mediated by TAAR1 (Sukhanov et al., 2014).

In view of the structural similarity between amphetamine-like compounds and β-PEA, it is not surprising that amphetamine and some amphetamine derivatives, including methamphetamine, MDMA, and 2-amino, 1-[2,5-dimethoxy-4-iodophenyl]-propane (DOI), are direct agonists at TAAR1. Their ability to stimulate cAMP production has been consistently reported at mouse, rat, rhesus monkey, and human TAAR1 expressed in various model cell systems, with potencies comparable to, or slightly weaker than, β-PEA or tyramine, although species differences and isomer-stereoselectivity in agonistic potency and efficacy were also noticed (Bunzow et al., 2001; Miller et al., 2005; Reese et al., 2007; Wainscott et al., 2007; Barak et al., 2008). Drugs in the amphetamine-class are well-known for their ability to increase extracellular levels of monoamines in the brain mainly by acting as monoamine transporter substrates, competing with monoamines for reuptake, causing transporter internalization, and promoting transporter-mediated efflux (reverse transport) (Khoshbouei et al., 2003, 2004; Elliott and Beveridge, 2005; Han and Gu, 2006). They also interfere with vesicular monoamine transporter-2, depleting monoamine vesicular storage, and increasing cytosolic monoamine availability for reverse transport (Brown et al., 2002; Partilla et al., 2006; Lizarraga et al., 2015; Freyberg et al., 2016). The potent DA-releasing capacity of these psychostimulants critically underlies their reinforcing properties and abuse liability (Marona-Lewicka et al., 1996; Howell and Kimmel, 2008). The magnitude of amphetamine-induced DA release in the ventral striatum has been positively correlated with subjective experience of euphoria (Drevets et al., 2001) and self-reported drug-wanting in humans (Leyton et al., 2002). In this regard, the identification of TAAR1 as a novel target for amphetamine and related drugs reveals a previously unrecognized mechanism of action for stimulant drugs and brings about a new opportunity for developing efficacious anti-addiction agents. Studies using transfected cells and rhesus monkey and mouse brain striatal synaptosomes showed that methamphetamine activation of TAAR1 triggers a series of cellular phosphorylation cascades, leading to reduced DA uptake, enhanced DA efflux, and DAT internalization, which partially contribute to the known DA-releasing effects of methamphetamine (Xie and Miller, 2009a). These actions of methamphetamine at TAAR1 suggest that pharmacological modulation of TAAR1 may hold promise as a therapeutic tool in methamphetamine addiction. Amphetamines possess therapeutic value themselves although their abuse potential strongly limits their long-term efficacy. Amphetamine is used as a treatment for ADHD, which has been associated with impaired monoaminergic modulation of prefrontal cortical function (Bymaster et al., 2002; Arnsten, 2006) and for narcolepsy and obesity, which are also associated with deficits in DA function (Mefford et al., 1983; Mitler and Hajdukovic, 1991; Wang et al., 2001; Geiger et al., 2009). The cognitive-enhancing, anti-narcoleptic, and anorexigenic effects of amphetamine have been attributed to its ability to increase monoamine transmission (Nishino et al., 1997; Wang et al., 2001; Wisor et al., 2001; Arnsten, 2006). The discovery that amphetamine-like compounds activate TAAR1 raises the possibility that TAAR1 may serve as target for therapeutic intervention in these neurological and psychopathological conditions, especially taking into consideration that TAAR1 can exert direct control over DA function, as described in the preceding sections.

Finally, an interesting finding is that several efficacious ligands at TAAR1 are known as antagonists at biogenic amine receptors or inhibitors of monoamine transporters. For example, the adrenergic or serotonergic antagonists phentolamine, tolazoline, and cyproheptadine, and the non-substrate DA transporter inhibitors, nomifensine, and 1-methyl-4-pheny1-1,2,3,6-tetrahydropyridine (MPTP), are all agonists at TAAR1 (Bunzow et al., 2001). Consequently, the identification of TAAR1 as an action site for these compounds may complement our understanding of their better recognized antagonistic effects at other GPCR or transporter proteins. By contrast, cocaine, a DAT inhibitor and potent psychomotor stimulant, exhibited low affinity at TAAR1 (Bunzow et al., 2001).

## TAAR1 and neuropsychiatric disorders

The implications of TAAR1 in neuropsychiatric disorders are wide-ranging and are summarized in Table 1. PD is characterized by a progressive degeneration of dopaminergic cells along the nigrostriatal pathway and subsequent loss of DA in the striatum (Lotharius and Brundin, 2002). L-DOPA treatment remains the gold standard pharmacotherapy for PD due to its ability to partially replenish striatal DA levels. However, chronic L-DOPA treatment produces disabling motor side effects such as dyskinesias and motor fluctuations (Lloyd et al., 1975; Dauer and Przedborski, 2003). Given, the ability of TAAR1 to downregulate DA transmission, medications that suppress TAAR1 activation may hold promise for treating PD. Supporting this hypothesis, the effectiveness of L-DOPA in reducing parkinsonian symptoms was enhanced in TAAR1-deficient mice compared to their wild-type counterparts (Sotnikova et al., 2008). Moreover, upregulation of the β-arrestin2 pathway has been associated with reduced levels of L-DOPA-induced dyskinesia, with no concomitant decreases in the therapeutic efficacy of L-DOPA (Urs et al., 2015). As discussed above, this signaling pathway is modulated by TAAR1, further strengthening the idea that TAAR1-based pharmacotherapies might have implications for the treatment of PD.

**Table 1:** **Implications of TAAR1 in major neurological diseases and psychopathological disorders**.

**Neurological conditions**	**Major findings**	**Studies**	**Implication**
Parkinson's disease (PD)	1. Therapeutic effectiveness of L-DOPA was enhanced in TAAR1-deficient mice	Sotnikova et al., 2008	TAAR1 antagonists or partial agonist may aid in L-DOPA treatment for PD
	2. β-arrestin 2 pathway signaling is associated with reduced L-DOPA-induced dyskinesia; TAAR1 activation modulates this pathway	Espinoza et al., 2015; Harmeier et al., 2015; Urs et al., 2015	
Psychosis	1. All human TAAR genes are clustered in a chromosome region associated with bipolar disorder and schizophrenia	Borowsky et al., 2001; Bunzow et al., 2001	TAAR1 deficiency may contribute to the etiology and neuropathology of psychosis, in particular schizophrenia. TAAR1 agonists may be effective in alleviating psychotic symptomatology
	2. TAAR1 KO mice had elevated brain high-affinity D2s receptors and showed dopaminergic supersensitivity, resembling patients with schizophrenia	Wolinsky et al., 2007	
	3. Patients with schizophrenia showed reduced striatal D-neurons, which might lead to reduced TA synthesis and decreased TAAR1 stimulation	Ikemoto et al., 2003	
	4. TAAR1 KO mice showed altered structure and function of cortical NMDA receptors, accompanied with behavioral perseveration and impulsivity; TAAR1 full, and partial agonists reversed pharmacologically- or genetically-induced glutamatergic hypofunction and reduced impulsivity in normal mice, consistent with a hypoglutamatergic hypothesis of schizophrenia	Revel et al., 2011, 2012b, 2013; Espinoza et al., 2015	
	5. TAAR1 KO mice had impaired sensorimotor gating, a key characteristic of schizophrenia reflecting dysfunction of dopaminergic and glutamatergic pathways	Wolinsky et al., 2007	
	6. TAAR1 agonists produced brain activation patterns similar to those elicited by olanzapine in rodents	Revel et al., 2013	
	7. Increased signaling of GSK3β has been linked to schizophrenia, while TAAR1 interacts with D2 receptors to shift β-arrestin 2 recruitment from D2 receptor to TAAR1, resulting in reduced GSK3β activity	Harmeier et al., 2015	
Drug addiction	1. TAAR1 KO mice had elevated spontaneous firing rate of midbrain DA neurons and increased extracellular DA in the NAc. They also showed enhanced sensitivity to the monoamine-releasing and locomotor-stimulating effects of amphetamine and MDMA, and to methamphetamine-induced conditioned place preference	Wolinsky et al., 2007; Lindemann et al., 2008; Di Cara et al., 2011; Leo et al., 2014; Achat-Mendes et al., 2012	TAAR1 is constitutively active or tonically activated by ambient levels of amines and controls DA activity, therefore TAAR1 activation level by full or partial agonists may regulate the up and downs of the DA system during the addiction cycle. Moreover, both partial and full activation of TAAR1 is effective in reducing abuse-related behavioral and neurochemical effects of cocaine and methamphetamine, supporting TAAR1-based pharmacotherapy in addiction
	2. While TAAR1 full agonists reduced the firing frequency of midbrain DA neurons in mice brain slices, a TAAR1 antagonist and TAAR1 partial agonists increased it	Bradaia et al., 2009; Revel et al., 2011, 2012b, 2013	
	3. A TAAR1 full agonist reduced electrically evoked DA release in the dorsal striatum and NAc in mice brain slices whereas a TAAR1 antagonist increased NAc DA release	Leo et al., 2014	
	4. TAAR1 partial agonists prevented cocaine-induced DA overflow and transiently blocked methamphetamine-evoked DA accumulation in the NAc of rats	Pei et al., 2014; Cotter et al., 2015	
	5. In rodents, selective TAAR1 partial and full agonists reduced amphetamine- and cocaine-induced hyperlocomotion; decreased cocaine-induced behavioral sensitization, conditioned place preference, self-administration, the motivation to take cocaine, and relapse of cocaine seeking; downwardly shifted full dose-response curve for cocaine self-administration; and prevented cocaine-induced potentiation of brain reward function	Revel et al., 2011, 2012b, 2013; Thorn et al., 2014; Pei et al., 2014, 2015	
6. In rodents, TAAR1 partial agonists reduced methamphetamine-induced behavioral sensitization, self-administration, and relapse of methamphetamine seeking	Jing et al., 2014, Cotter et al., 2015		
Affective disorder	1. TAAR1 agonists enhanced performance in differential reinforcement of low-rate behavioral paradigm in monkeys and reduced immobility time in forced-swim test in rats	Revel et al., 2012b, 2013	TAAR1 may constitute a novel target for medicinal development for depression and anxiety disorders
	2. TAAR1 agonists prevented stress-induced hyperthermia in mice, indicating anxiolytic effects	Revel et al., 2011, 2012b	
Others	1. TAAR1 partial and full agonists improved response accuracy in object retrieval task in monkeys and reduced PCP-induced decline in attentional set-shifting in rats	Revel et al., 2012b, 2013	TAAR1-based ligands may have wider benefits beyond disease conditions, such as enhancing cognition and wakefulness
	2. In monkeys, selective TAAR1 agonist increased wakefulness, with a potency similar to caffeine, but did not have caffeine-related side effects	Revel et al., 2012b, 2013	

Several lines of evidence have implicated TAAR1 in the etiology of psychosis. First, all human TAAR genes are tightly clustered in a narrow region of 109 kb of chromosome 6q23.1 (Borowsky et al., 2001; Bunzow et al., 2001; Lindemann et al., 2005), which is close to or among a few susceptible loci that have been reproducibly associated with schizophrenia (Straub et al., 1995; Cao et al., 1997; Lindholm et al., 1999) and bipolar affective disorder (Rice et al., 1997; Dick et al., 2003; Sklar et al., 2008) in linkage or association studies, suggesting a possible causal role of this receptor family in the etiology of psychosis/polygenic neuropsychiatric disorders. Consistent with this notion, TAAR1 knock-out mice exhibited an elevation of brain high-affinity D2s receptors and dopaminergic supersensitivity (Wolinsky et al., 2007). In patients with schizophrenia, increased availability and excessive stimulation of D2s have been observed during a psychotic episode (Laruelle et al., 1996; Abi-Dargham et al., 1998). Further, the effectiveness of antipsychotics to occupy D2s has been linked to clinical responsiveness and extrapyramidal side effects (Pickar, 1995; Kapur et al., 2000; Seeman, 2011). Also, greater response to DA stimulants, such as amphetamine and methylphenidate, has been found in schizophrenics compared to healthy controls (Seeman, 2011). These findings reveal common characteristics of altered dopaminergic activity in TAAR1 deficient mice and schizophrenia patients, suggesting that abnormal TAAR1 function may contribute to psychotic symptoms. In addition, dysregulation of the β-arrestin2/AKT/GSK3β pathway has been implicated in schizophrenia and a wide range of clinically effective antipsychotics were shown to antagonize D2 receptor-mediated β-arrestin2 signaling and inhibit GSK3β activity (Masri et al., 2008; Beaulieu et al., 2009; Emamian, 2012; Urs et al., 2012). Therefore, the finding that TAAR1 stimulation through the β-arrestin2-dependent pathway leads to a silencing of GSK3β adds further support for the involvement of TAAR1 in schizophrenia and suggests that drugs that increase β-arrestin2 signaling by TAAR1 may be effective treatment for this disease. In addition, a novel D-neuron hypothesis has recently emerged proposing the implication of the TA system in the pathogenesis of schizophrenia. D-neurons are non-monoaminergic AADC-containing striatal cells, postulated to be TA-producing neurons (Keiko, 2012; Ikemoto, 2013). According to this hypothesis, decreased proliferation of neural stem cells in the subventricular zone of the lateral ventricle leads to a reduction of striatal D-neurons and thus insufficient TA synthesis. The ensuing decrease in TAAR1 stimulation ultimately causes augmented DA release in the NAc and striatal DA hyperstimulation further inhibits the proliferation of the neural stem cells, further accentuating DA hyperactivity (Ikemoto, 2015). However, this proposition is mainly built upon the observation of reduced striatal D-neurons in patients with schizophrenia (Ikemoto et al., 2003) and requires further experimental support. Apart from dysfunctional dopaminergic activity, reduced glutamatergic transmission has also been proposed as a causal factor in schizophrenia, particularly in relation to the negative symptoms and cognitive impairment associated with the disease (Javitt, 2004; Coyle, 2006; Goff and Coyle, 2014). The NMDA receptor antagonist, PCP, has been widely used in rodent models of schizophrenia to produce psychotic symptoms and some atypical antipsychotics are known to modulate NMDA-mediated neurotransmission (Jentsch and Roth, 1999; Goff and Coyle, 2014). Interestingly, selective activation of TAAR1 by both full and partial agonists has been shown to reverse glutamatergic hypofunction induced by selective NMDA receptor antagonists in wild-type animals and mutant mice with NMDA receptor deficiency (Revel et al., 2011, 2012a, 2013), suggesting that TAAR1 activation may enhance glutamatergic function. Moreover, TAAR1 KO mice displayed altered subunit composition of cortical NMDA receptors and dysregulation of NMDA receptor-dependent synaptic function, accompanied by increased perseveration and impulsivity. Moreover, full and partial activation of TAAR1 by specific agonists reduced impulsive behaviors in normal mice, suggesting a facilitating role of TAAR1 on cortical glutamate transmission and associated behavioral functions (Espinoza et al., 2015). In addition, TAAR1 KO exhibited significant deficits in sensorimotor gating measured as impaired prepulse inhibition of acoustic startle (Wolinsky et al., 2007), which is a known behavioral signature in schizophrenia regulated by both dopaminergic and glutamatergic mechanisms (Wan et al., 1995; Wan and Swerdlow, 1996; Geyer et al., 2001). Also, the selective full and partial TAAR1 agonists, RO5256390 and RO5263397, respectively, produced a brain activation pattern akin to that induced by the antipsychotic drug, olanzapine (Revel et al., 2013). Together, these data persuasively implicate TAAR1 in the etiology and neuropathology of psychosis, and support the investigation of TAAR1-based pharmacological tools as treatments for this condition.

One of the best documented findings in TAAR1 pharmacology is the ability of this receptor to regulate the neurochemical and behavioral actions of psychomotor stimulant drugs, which has fuelled the notion that TAAR1-selective compounds could have therapeutic value in addiction treatment. Psychostimulant drugs exert their effects in the CNS mainly by causing strong alterations in DA transmission and long-lasting neuroadaptations in the dopaminergic system (Volkow et al., 1999; Grace, 2000; Pierce and Kumaresan, 2006). Indeed, the close anatomical and functional interaction of TAAR1 with the DA system and the documented role of TAs in brain reward and reinforcement functions are consistent with this hypothesis. Moreover, as discussed in the previous sections, studies with transgenic mice and selective agonists at TAAR1 have provided compelling demonstration of TAAR1's ability to downregulate the monoaminergic response to psychomotor stimulants. Several recent studies have been aimed at investigating the therapeutic potential of TAAR1 agonists in well-validated animal models of drug addiction. Revel and collaborators provided the first evidence that the partial agonist, RO5203648, decreased cocaine-induced locomotor activity and self-administration in rats (Revel et al., 2012b). Subsequent work revealed that the partial and full agonists, RO5203648 and RO5256390, respectively, both produced downward shifts in the dose-response curve for cocaine self-administration and prevented cocaine-induced lowering of ICSS thresholds (Pei et al., 2015), indicating that partial or full TAAR1 activation blocks the reinforcing properties of cocaine. In agreement with these observations, another TAAR1 partial agonist, RO5263397, reduced cocaine-induced behavioral sensitization and cocaine conditioned place preference (Thorn et al., 2014). Importantly, TAAR1 agonists also prevented context-induced cocaine relapse and cue- and cocaine prime-induced reinstatement of cocaine seeking (Pei et al., 2014; Thorn et al., 2014). In the case of methamphetamine, the partial agonists, RO5203648, and RO5263397, reduced methamphetamine-induced behavioral sensitization, and self-administration (Jing et al., 2014; Cotter et al., 2015). RO5263397 also blocked cue- and drug prime-induced reinstatement of methamphetamine seeking (Jing et al., 2014). Remarkably, at doses that were effective at attenuating drug-related behaviors, such as drug relapse and reinstatement, these agonists did not suppress responding maintained by natural reinforcers, ruling out non-specific effects of TAAR1 agonists on general motoric and motivational functions (Pei et al., 2014; Cotter et al., 2015). In fact, using a progressive ratio schedule of reinforcement, the partial agonist, RO5203648, decreased the break point for cocaine self-administration but enhanced responding for food (Pei et al., 2014), suggesting clearly dissociable effects of TAAR1 activation on drug- and food-maintained responding. Although, the mechanisms by which TAAR1 activation decreases motor stimulant effects are not well-understood, using fast-scan cyclic voltammetry we have shown previously that activation of TAAR1 with the partial agonist, RO5203648, prevented the potentiation of DA transmission caused by cocaine in the NAc without affecting DA uptake kinetics. This suggests that the TAAR1 agonist attenuated cocaine-stimulated DA overflow by mechanisms other than direct interference with DA uptake (Pei et al., 2014). Together, these findings demonstrate that TAAR1 agonists are effective at modulating the neurochemical and behavioral effects of psychomotor stimulants and lend strong support to the development of medications targeting TAAR1 as treatments for psychostimulant addiction. Furthermore, other classes of abused substances such as opiates, ethanol, cannabinoids, and nicotine all have been shown to directly or indirectly influence DA transmission. Although, DA-independent processes are also implicated in the psychoactive actions of addictive drugs (Tanda et al., 1997; Pierce and Kumaresan, 2006; Hiranita et al., 2013; Söderpalm and Ericson, 2013), the ability of TAAR1 to modulate DA transmission still strongly raises the intriguing possibility that the TAAR-based approach will form a new generation of medications for addictive disorders.

TAAR1 may also play a crucial role in emotional regulation given that TAAR1 expression is enriched in the amygdala (Borowsky et al., 2001), a key integrative region for emotions, emotional behavior, and motivation (Phelps and LeDoux, 2005). The association between TA deficiency and affective disorders, such as depression, suggests that TAAR1 activation may serve to stabilize maladaptive mood and emotional fluctuations and contribute to the effects of antidepressants or anxiolytics. In support of this hypothesis, TAAR1 agonists were shown to enhance performance in a differential reinforcement of low-rate behavioral paradigm in monkeys, reduce immobility time in a forced-swim test in the rat, and prevent stress-induced hyperthermia in mice, thus demonstrating anti-depressant and anxiolytic properties (Revel et al., 2011, 2012a, 2013).

Recent evidence indicates that pharmacological manipulation of TAAR1 could have wider applications to encompass not only disease treatment but also mental health promotion. TAAR1 selective ligands have been shown to enhance cognition and increase wakefulness (Revel et al., 2011, 2012a, 2013). Several selective full and partial agonists at TAAR1 improved response accuracy in an object retrieval task in monkeys and rescued PCP-induced decline in attentional set-shifting in the rat, demonstrating pro-cognitive effects including increased attention, adaptive response inhibition, and cognitive flexibility (Revel et al., 2012a, 2013). Also, two selective TAAR1 partial agonists increased wakefulness in a manner similar to caffeine without eliciting caffeine-associated side effects such as increased locomotion and changes in core body temperature (Revel et al., 2012a, 2013). Collectively, these findings suggest that TAAR1 pharmacology may have implications for cognitive enhancement, sleep control, and mental health advancement in a broader context that concerns workplace performance and general well-being.

## Conclusions

The novel findings discussed in this review uncover the pivotal role of TAs and their associated receptors in the functional regulation of classical aminergic systems and highlight the relevance of TAAR1 as a molecular target to engineer innovative forms of treatment for neurological disease. Although, the physiological significance of TAs was not fully recognized until the discovery of their receptor family, TAs have long been associated with the classical amines. Neuropharmacological research into TAAR1, being the only member of the TAAR family that is phylogenetically conserved in mammals, has gained considerable momentum over the past decade, largely fuelled by the development of specific tools to unveil its neurophysiological and behavioral functions, most notably transgenic models, and direct ligands. TAAR1 interacts with the DAT and DA D2 receptors, modulates DA neuron firing rate and DA release, regulates the biochemical signaling cascades triggered by amphetamine-like psychostimulants and, when selectively deleted or activated, has the remarkable ability to influence key behaviors associated with neurological dysfunction. As a result, there is growing interest in developing TAAR1-based therapies for the treatment of a range of neuropsychiatric disorders, including PD, psychosis, drug addiction, and mood disorders. Besides, the demonstrated wake-promoting and cognitive-enhancing properties of TAAR1 agonists may provide a platform for the design of complementary interventions for other forms of maladaptive behaviors. However, our understanding of the underlying mechanisms by which TAAR1 is able to exert such notable regulatory effects remains scant and future efforts should be devoted to fully elucidate the molecular pathways and downstream targets that are recruited by TAAR1 under a variety of physiological and non-physiological conditions. Moreover, the development of more refined research tools such as TAAR1 agonists and antagonists, particularly the latter, with enhanced pharmacokinetic/dynamic profile will allow more effective assessment of TAAR1 as a molecular target for the development of therapeutics in neuropsychiatry. Despite, these limitations, compelling evidence now links TAs and TAAR1 to neuropsychiatric disorders and, as further knowledge is unveiled and better research tools become available to study TAAR1, we can look forward to a new era of basic and translational neuroscience research.

## Author contributions

All authors listed, have made substantial, direct and intellectual contribution to the work, and approved it for publication.

### Conflict of interest statement

The authors declare that the research was conducted in the absence of any commercial or financial relationships that could be construed as a potential conflict of interest.
